# Development of a Machine Learning–Based Patient Classification System for a Gastroenterology Ward

**DOI:** 10.1155/jonm/2039402

**Published:** 2026-07-29

**Authors:** Hong-Fei Ren, Ming-Fang Wei, Ming Shen, Lei Lv, Yao Hu, You-Ping Li, Chang-Qing Liu, Yan Jiang

**Affiliations:** ^1^ Department of Gastroenterology, West China Hospital, West China School of Nursing, Sichuan University, Chengdu, Sichuan, China, scu.edu.cn; ^2^ Chengdu CloudStack Technology Co., Ltd., Chengdu, Sichuan, China, scu.edu.cn; ^3^ West China Biomedical Big Data Center, West China Hospital, Sichuan University, Chengdu, Sichuan, China, scu.edu.cn; ^4^ Chinese Evidence-Based Medicine Centre, Cochrane China Center, West China Hospital, Sichuan University, Chengdu, Sichuan, China, scu.edu.cn; ^5^ Trauma Center of West China Hospital, West China School of Nursing, Medicine and Engineering Interdisciplinary Research Laboratory of Nursing & Materials, West China Hospital, Sichuan University, Chengdu, Sichuan, China, scu.edu.cn; ^6^ Nursing Department of West China Hospital, West China School of Nursing, Sichuan University, Chengdu, China, scu.edu.cn

**Keywords:** gastroenterology ward, machine learning, nursing staff allocation, patient classification system

## Abstract

**Background:**

Patient classification systems (PCSs) are frequently used to estimate hours of nursing care to support budgeting and guide staffing decisions. However, despite a clear clinical need, there is as yet no validated and reliable PCS designed specifically for gastroenterology wards.

**Objective:**

To develop a PCS tailored to gastroenterology wards that can accurately quantify nursing workload and support evidence‐based nurse staffing decisions.

**Methods:**

This study used Orem’s self‐care theory and Henderson’s human needs theory as a foundation and applied machine learning techniques to construct a PCS for a gastroenterology ward in a general tertiary hospital. Patients were enrolled using convenience sampling, and information on their demographic characteristics, together with daily nursing activities and frequencies, was retrospectively extracted from the hospital information system (HIS) between July 1, 2019, and June 30, 2020. A workload measurement method was used to calculate the amount of nursing care received by each patient over a 24‐hour period, thereby forming the study database. A decision tree model was developed to classify patients. The PCS was subsequently refined and validated using a prospective observational study of 357 patients conducted from December 1, 2022, to March 31, 2023.

**Results:**

The final PCS included two primary categories and five subcategories, each of which was defined by specific patient characteristics and their corresponding 24‐hour nursing time requirements: Category 1: “Day of surgery and first postoperative day,” in which patients were subdivided into three levels based on surgical complexity, self‐care capacity, and illness severity (Surgery 1, Surgery 2, and Surgery 3), requiring 1.66, 2.82, and 4.15 nursing hours per 24 h, respectively, and Category 2: “Other days” patients, who were subdivided into two groups based on critical illness, self‐care ability, and disease severity (Category 1 and Category 2), with each requiring 0.96 and 3.42 nursing hours per 24 h, respectively. Internal and external validation demonstrated good model fit and acceptable predictive performance.

**Conclusions:**

Patients could be rapidly classified using defined indicators, enabling accurate prediction of their 24‐hour nursing care requirements. The PCS exhibited strong internal consistency, stability, and generalizability, thereby supporting the reliability of its results.

**Implications for Nursing Management:**

This PCS provides a scientific basis for nurse staffing decisions and may support hospital administrators and health authorities in the development of data‐driven nurse workforce allocation policies.

## 1. Introduction

The global nursing workforce is experiencing a critical imbalance between supply and demand. According to the *State of the World’s Nursing 2025* report [1], there was a worldwide shortage of approximately 5.8 million nurses in 2023. Nurse staffing levels are a key determinant of healthcare quality and patient safety, as they directly affect the timeliness, effectiveness, and personalization of care delivery [2]. However, the appropriate allocation of nursing staff remains challenging as patient needs vary widely depending on diagnosis, treatment intensity, and disease severity. Consequently, there is an urgent need for scientific methods of patient stratification that can identify differences in care requirements, quantify nursing workloads, and guide rational staff deployment. Patient classification systems (PCSs) categorize patients according to the intensity of care required, clinical acuity, and resource utilization, thereby providing standardized and objective measures of nursing demand [3]. By enabling data‐driven workforce allocation, PCSs help ensure that staffing levels are aligned with real‐time patient needs [4, 5]. A well‐designed PCS can improve the efficiency of nursing resource utilization while also enhancing care quality, reducing clinical errors, and increasing patient satisfaction, making it an essential component of modern hospital management.

Globally, numerous PCSs have been developed and applied in clinical practice. These systems generally fall into three types: prototype‐based, factor‐based, and hybrid systems. Hybrid PCSs, which combine the objectivity and precision of factor‐based approaches with the simplicity and usability of prototype systems, are increasingly recognized as the preferred model for nursing workforce management [6]. Evidence suggests that implementation of a PCS for nurse staffing reduces workload imbalance, nurse burnout, and the likelihood of adverse events, while improving both nurse job satisfaction and productivity. At the organizational level, optimized staffing can also reduce operational costs and improve economic efficiency [4, 7].

Despite these advances, many existing PCSs lack a strong theoretical foundation, use poorly defined patient indicators, and have not undergone rigorous validation in real‐world staffing contexts, all of which limit their accuracy, consistency, and practical application. Additionally, existing PCSs mostly rely on static or empirical indicators, lacking the data‐driven integration capability of real‐time disease dynamics and the complexity of care. This gap in reality results in poor predictive validity of the models, making it difficult to precisely match care needs with human resource supply and constraining the scientific nature and clinical adaptability of resource allocation. As synergy between clinical nurse staffing and data‐driven models is a key element in improving the quality and efficiency of healthcare systems [8], the development of a data‐driven PCS model for nursing staff allocation is not only operational but also strategic. This problem is particularly evident in gastroenterology wards, where patients present with a wide range of conditions ranging from acute gastrointestinal bleeding to chronic liver disease, leading to highly variable nursing care needs. The absence of a specialized and validated PCS for gastroenterology settings constrains efficient nurse staffing and limits improvements in care quality and efficiency.

To address these considerations, this study aimed to develop a hybrid PCS specifically for gastroenterology wards based on Henderson’s human needs theory [9] and Orem’s self‐care theory [10]. The human needs theory identified 14 basic human needs and assists in the comprehensive classification of the needs of hospitalized patients and provides a theoretical basis for determining nursing programs and improving the scientific rigor and accuracy of measuring nursing workload. The Orem self‐care theory aims to maintain and promote the self‐care ability of patients, thereby aligning with the objective of this study to explore the nursing services and human resource differences required by patients with different levels of self‐care. This theory can guide the accurate assessment of patients’ nursing needs and the selection of indicators reflecting self‐care ability and at the same time provide a scientific basis for identifying the underlying influences of patient factors on nursing workload. Furthermore, multiple algorithms, including decision trees (DTs), random forests (RFs), and support vector machines (SVMs), were used to identify key predictors of patient acuity and to construct a robust classification framework. The resulting PCS is designed to provide highly accurate classification while remaining practical and easy to use in routine clinical workflows. Ultimately, this study seeks to provide a scientifically grounded and operationally feasible tool to support nursing workforce planning in gastroenterology wards and to advance evidence‐based healthcare management.

## 2. Methods

**FIGURE 1:**
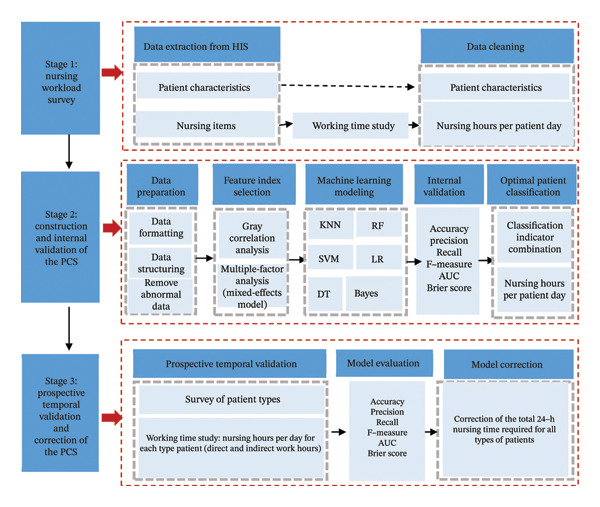
Technical roadmap for developing the PCS.

This study was conducted in three sequential stages. Stage 1 (nursing workload survey): Guided by Orem’s self‐care theory and Henderson’s human needs theory, a combination of retrospective data extraction and direct observation was used to calculate the duration of nursing received by each patient during a 24‐hour period in the gastroenterology department. Stage 2 (PCS development): A gastroenterology‐specific PCS was constructed using machine learning algorithms. Stage 3 (PCS validation and refinement): The system was externally validated and adjusted through prospective observation. The overall study design is illustrated in Figure 1. The study adhered to the Reporting of Observational Studies in Epidemiology (STROBE) guidelines. Ethical approval was obtained from the hospital’s Research Ethics Committee (No. 2021‐444), and informed consent was secured from all participants prior to inclusion.

### 2.1. Nursing Workload Survey

A retrospective database review combined with a time‐and‐motion observational study was conducted to quantify the 24‐hour nursing workload for each patient in the gastroenterology ward of a large tertiary general hospital in China.

First, information on all hospitalized patients and their associated nursing activities between July 1, 2019, and June 30, 2020, were retrospectively retrieved from the hospital electronic health record (EHR) system using purposive sampling. Patients with interrupted hospitalization (e.g., those transferred out and subsequently readmitted during the same hospitalization) were excluded. The extracted data included patient identifiers (registration number, name, sex, and age), diagnoses, comorbidities, surgical procedures and levels, daily vital signs (temperature, blood pressure, pulse/heart rate, respiratory rate, and SpO_2_), level of consciousness, diet, nursing level, critical status, illness severity, and self‐care ability throughout hospitalization, as well as all diagnoses and treatment activities related to nursing care.

Second, a direct observational survey was conducted to determine the mean duration of each nursing activity. The duration of each activity was timed seven times by two trained observers using stopwatches. The minimum number of measurements required for each activity was calculated using the formula $n=\left(\mu_{\alpha }^{2}\sigma^{2}\right)/\delta^{2}$ [11], where *σ* was 98.5 s (derived from a pilot study of intravenous infusion), *δ* was set at ≤ 60 s, and *α* was 0.05 for a two‐sided test. All observers received standardized training on the study objectives, definitions, measurement procedures, and quality control requirements. A premeasurement phase was conducted to ensure consistency among observers.

Finally, all extracted data were cleaned and processed in a MySQL database using structured query language (SQL). This yielded daily patient‐level characteristic indicators and the frequencies of all nursing activities. Nursing hours per patient day (NHPPD) is the most frequently used parameter and is considered to be applied more effectively as a nurse staffing measure [12], with an intraclass correlation coefficient (ICC) ranging from 0.691 to 0.998 [13, 14]. The NHPPD was calculated using the following equation:

(1) NHPPD=∑k=1nKi∗Ti,

where *K* represents the frequency of a given nursing activity, *T* represents the time required for that activity, and *n* is the number of distinct nursing activities.

### 2.2. Development of a Gastroenterology‐Specific PCS

#### 2.2.1. Data Cleaning and Processing

The analytical dataset included 30,410 records, with each record representing one patient along with corresponding demographic and clinical characteristics and the NHPPD value. All data were standardized, structured, and screened for outliers before the application of machine learning methods. Specifically: (1) surgical procedures were classified into four levels according to size, anatomical location, risk, and technical complexity, following the national classification directory for digestive endoscopy and vascular interventional procedures issued by the National Health Commission of China [3, 4]; (2) disease diagnoses were coded based on the ICD‐10 coding system; (3) nursing care levels were categorized as special level or Levels 1–3 in accordance with the “Guidelines for Graded Care in General Hospitals (Trial)” published by the Chinese Ministry of Health (http://www.nhc.gov.cn/yzygj/s3593/200905/bc4b8bab01d146b8a024fad4746854eb.shtml) [15]; (4) patient self‐care ability was evaluated using the Barthel Index; the scores were classified as follows: 100 (independent), 61–99 (mild dependence), 41–60 (moderate dependence), and ≤ 40 (severe dependence) [16]; and (5) disease severity was assessed using the Disease Severity Evaluation Scale developed by Ding Junqin et al. [17] (Table 1), with total scores defining four categories: critical (≥ 12), severe (7–11), moderate (2–6), and mild (0‐1). Because nursing hours on the day of discharge were recorded as zero and did not meaningfully reflect patient care requirements, these data were considered abnormal and were excluded from the analysis.

**TABLE 1:** Disease Severity Evaluation Scale.

Items	Scores				
	0	1	2	3	4
*T* (°C)	36.0–37.0	37.1–37.5 OR 35.5–35.9	37.6–38.9 OR 35.0–35.5	39.0–39.9 OR < 35.0	≥ 40.0
SBP (mmHg)	89–139	140–159	160–179 OR 85–89	≥ 180 OR 80–84	≥ 190 OR < 80
*R* (per minute)	12–20	21–25 OR 10–11	26–35	> 35 OR 6–9	> 50 OR ≤ 5
*P*/HR (per minute)	60–90	91–100 OR 51–59	101–140 OR 41–50	141–160 OR 30–40	> 160 OR < 30
SpO_2_ (%)	≥ 95	90–94	85–90	80–85	< 80
Consciousness	Awake	—	Hypersomnia	Haziness	Coma
Dietary intake	Normal	Eat less	—	Aphagosis	—
Age (years old)	—	61–70	71–84	85–89	≥ 90
Disease status	Recovery phase of diseases	Chronic disease OR injured	Acute disease OR injured	—	—
Complicating diseases (number)	0	1	2	3	4

#### 2.2.2. Development of the PCS

The data collected in Section 2.2.1 were randomly divided into a training set and a testing set at a ratio of 7:3. To address the issue of repeated measurements, the split was performed at the level of patient ID, thereby ensuring that all data points from the same patient were assigned exclusively to either the training set or the testing set. The training dataset was used for the development of the primary PCS, and the testing dataset was used for internal validation of the PCS model. The PCS was developed using a standard machine learning workflow, as follows. (1) Exploratory data analysis: The Matplotlib module in Python was used to visualize the distribution of 24‐hour nursing workload, and descriptive and inferential statistics were applied to characterize data patterns. (2) Feature selection: Gray correlation analysis combined with a mixed‐effects model was applied to identify the most informative patient characteristics for patient classification. (3) Hyperparameterization [18]: Based on prior studies showing that PCS models often rely on integer‐based divisions of nursing hours, workload thresholds were defined according to the observed distribution. Patients on the day of surgery and the first postoperative day were classified into four workload groups (≤ 1 h, 1‐2 h, 2‐3 h, and > 3 h), while patients on other days were divided into two groups (≤ 2 h and > 2 h). Furthermore, we introduced a hyperparameter tuning mechanism that combines random search with stratified K‐fold cross‐validation. Specifically, this included the number of trees (100–800), the maximum depth (3–7), the learning rate (sampled continuously within the range of 0.01–0.3), the row sampling ratio and column sampling ratio (both sampled within the range of 0.6–1.0), and the L1 and L2 regularization strength, as well as the minimum subnode weight and the minimum gain required for tree splitting. Five‐fold stratified cross‐validation was used to ensure that the proportion of each category in each fold was consistent with the overall distribution. (4) Management of overfitting: A three‐in‐one overfitting management system consisting of “learning curve diagnosis + early stopping loss curve monitoring + regularization strength effect analysis” was established. This involved (1) learning curve diagnosis in which the weighted F1 curve of training/validation was plotted against increasing sample size. A final difference between the two of greater than 0.10 was considered overfitting, leading to the triggering of a parameter adjustment suggestion. (2) Iterative loss curve: The tree number was fixed at 1000, and the multiclass logarithmic loss of training/testing was monitored. The optimal iteration round was automatically recorded when the test loss stopped decreasing or started to rise. (3) Regularization analysis: The gap in training and cross‐validation accuracy was examined when the *λ* value ranged from 0.1 to 10. (5) Model training: Six machine learning algorithms, including logistic regression (LR), SVMs, k‐nearest neighbors (KNN), DT, RF, and Naïve Bayes, were implemented using the scikit‐learn library in Python Version 3.8.2. (6) Model testing: Model performance was evaluated using 5‐fold cross‐validation across six metrics: accuracy, precision, recall, F‐measures, receiver operating characteristic (ROC) curves, and calibration curves. Calibration was further assessed using the Brier score, which ranges from 0 to 1. The model with the best overall performance was selected to define the preliminary PCS and included profiles of the patient characteristics and the corresponding ranges of 24‐hour nursing workloads.

### 2.3. PCS Validation and Refinement

A prospective observational study was conducted to collect data related to the PCS model, representing the prospective temporal validation dataset, which was used for external validation and refinement of the PCS. A random sample of 357 patients admitted to the gastroenterology ward between December 1, 2022, and March 31, 2023, was enrolled. Patient characteristics and 24‐hour nursing workloads were collected prospectively, and the optimal PCS model was applied to assess classification accuracy and guide revisions.

#### 2.3.1. Sample Size

The required sample size was estimated using the guideline of 10–20 observations for each predictor variable [19]. In the previous stage, six patient categories were identified, each with three characteristic indicators, resulting in a total of 18 indicators. Therefore, the sample required a minimum of 180 and a maximum of 360 patients.

#### 2.3.2. Data Collection

Data were recorded using a standardized form that captured patient demographics, clinical characteristics, and shift‐based nursing activities (both direct and indirect) with their associated time expenditures. Patients were grouped according to room and assigned nurse (14 groups of 6‒7 patients each), and one group was randomly selected each day for observation. Trained observers measured the duration of each nursing activity using stopwatches. Completed forms were reviewed the following day to ensure their completeness and accuracy before data entry.

#### 2.3.3. Data Analysis

The collected dataset was reanalyzed using a DT algorithm to construct the patient classification model, following the evaluation framework described in Section 2.2.2. Patients were assigned to categories according to the PCS, and the corresponding 24‐hour nursing hours for each category were used as the predicted values. Actual nursing workloads were calculated by summing the time spent on all nursing activities for each patient. As the nursing hours did not show a normal distribution, predicted and observed NHPPD values for each patient category were summarized using the median and interquartile range. Differences between predicted and actual values were assessed using the paired Wilcoxon signed‐rank test. A two‐sided *p* value < 0.05 was considered significant. Model accuracy was evaluated by calculating the proportion of patients whose observed NHPPD fell within the predicted range for their assigned category, using the following formula: the prediction accuracy rate = ([the number of actual values within the prediction range]/[the number of observation cases for each category of patients]) ∗ 100%.

An accuracy of at least 70% was considered indicative of acceptable model performance. If no statistically significant difference was detected between the predicted and observed values, no adjustment was applied. However, when a significant discrepancy was identified, a correction coefficient (*α*) was calculated during external validation, and the predicted NHPPD ranges were revised to generate a PCS that better reflected real‐world clinical practice.

## 3. Results

### 3.1. Nursing Workload Distribution

**FIGURE 2:**
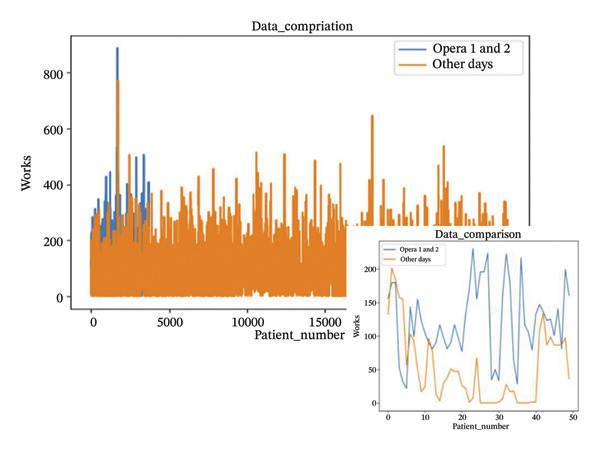
Distribution of nursing hours on the day of surgery and first postoperative day compared with other days.

The distribution of NHPPD for patients on the day of surgery and the first postoperative day differed markedly from that observed on all other hospital days (Figure 2). Statistical testing confirmed a significant difference between these two periods (*Z* = −38.799, *p* < 0.001; Table 2). Accordingly, the dataset was divided into two groups: “day of surgery and first postoperative day” and “other days.”

**TABLE 2:** Analysis of nursing hours on “the day of surgery and first postoperative day” compared with “other days.”

	Maximum value	Minimum value	Median	P25	P75	P75‐P25
The day of surgery and the first postoperative day	889.20	0.50	118.16	78.17	154.80	76.63
Other days	773.24	0.00	60.89	28.80	116.03	87.23

*Z*	−38.799					
*p*	< 0.001					

### 3.2. Identification of Key Patient Characteristics

For patients in the “day of surgery and first postoperative day” group, nursing workload was primarily influenced by surgical level, self‐care ability, and disease severity. In contrast, for patients hospitalized on “other days,” the major determinants of nursing time were whether the patient was critically ill, their level of self‐care, and severity of their illness (Tables 3 and 4).

**TABLE 3:** The mixed‐effects model for the nursing hours of patients on “the day of surgery and first postoperative day.”

Variables	Beta	*t*	*p*	95.0% CI	
				Lower limit	Upper limit
Constant	0.213	1.638	0.102	−0.042	0.468
Severity of illness (ref = 1)					
Severity of illness = 2	0.434	5.782	< 0.001	0.287	0.582
Severity of illness = 3	1.089	0.078	< 0.001	0.937	1.242
Severity of illness = 4	1.858	16.868	< 0.001	1.642	2.074
Self‐care ability (ref = 0)					
Self‐care ability = 1	0.462	6.694	< 0.001	0.327	0.597
Self‐care ability = 2	0.734	10.599	< 0.001	0.598	0.869
Self‐care ability = 3	1.122	16.006	< 0.001	0.984	1.259
Surgery level (ref = 1)					
Surgery level = 2	0.085	0.798	0.425	−0.124	0.295
Surgery level = 3	0.450	4.293	< 0.001	0.245	0.656
Surgery level = 4	0.497	4.692	< 0.001	0.289	0.705

*Note:* Severity of illness: 1 = mild, 2 = moderate, 3 = severe, and 4 = critical; self‐care ability: 0 = no dependence, 1 = mild dependence, 2 = moderate dependence, and 3 = severe dependence; surgery level: 1 = primary surgery; 2 = secondary surgery; 3 = tertiary surgery; 4 = quaternary surgery.

**TABLE 4:** The mixed‐effects model for the nursing hours of patients on “other days.”

Variables	Beta	t	*p*	95.0% CI	
				Lower limit	Upper limit
Constant	0.255	11.663	< 0.001	0.213	0.298
Severity of illness (ref = 1)					
Severity of illness = 2	0.141	5.992	< 0.001	0.0949	0.187
Severity of illness = 3	0.640	24.763	< 0.001	0.589	0.691
Severity of illness = 4	1.351	36.411	< 0.001	1.278	1.424
Self‐care ability (ref = 0)					
Self‐care ability = 1	0.226	15.146	< 0.001	0.197	0.255
Self‐care ability = 2	0.558	31.672	< 0.001	0.523	0.592
Self‐care ability = 3	1.150	63.739	< 0.001	1.114	1.185
Whether critically ill (ref = 0)					
Whether critically ill = 1	0.553	46.661	< 0.001	0.529	0.576

*Note:* Severity of illness: 1 = mild, 2 = moderate, 3 = severe, and 4 = critical; self‐care ability: 0 = no dependence, 1 = mild dependence, 2 = moderate dependence, and 3 = severe dependence; whether critically ill: 0 = no; 1 = yes.

### 3.3. Comparison of Machine Learning Model Performance

**FIGURE 3:**
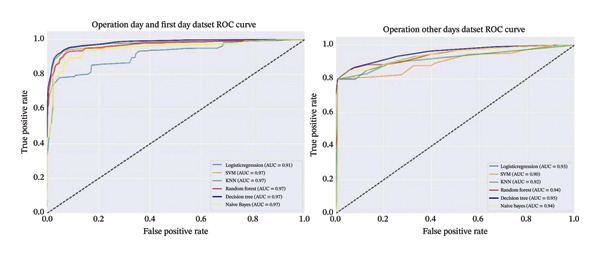
The ROC curve of the six models in “the day of surgery and first postoperative day” and “other days” classifications.

**FIGURE 4:**
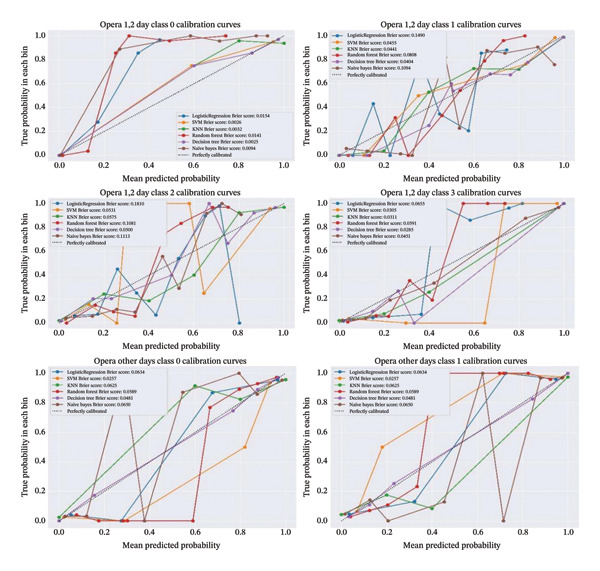
The Brier scores of the six models in “the day of surgery and first postoperative day” and “other days” classifications.

As shown in Tables 5 and 6, the DT algorithm demonstrated the strongest overall performance in both patient groups. For patients on the day of surgery and first postoperative day and for those on other days, the model achieved accuracies of 92.6% and 94.4%, AUC values of 0.980 and 0.950, and F‐measures of 0.926 and 0.944, respectively. These metrics indicate a high degree of classification reliability. Moreover, the ROC curves suggested that the DT model offered better predictive capability compared with the other algorithms examined (Figure 3). In addition, this model produced the lowest Brier scores across all scenarios (0.0025, 0.0404, 0.0500, 0.0285, 0.0481, and 0.0481) (Figure 4), indicating well‐calibrated performance.

**TABLE 5:** The performance evaluation results of the six models in the “the day of surgery and first postoperative day” classification.

Models	Accuracy	Precision	Recall rate	F‐measure	AUC (95% CI)
Decision tree	0.926	0.926	0.926	0.926	0.980 (0.982, 0.991)
SVM	0.926	0.926	0.926	0.926	0.970 (0.973, 0.984)
KNN	0.923	0.923	0.923	0.923	0.970 (0.970, 0.985)
Random forest	0.895	0.895	0.895	0.895	0.970 (0.959, 0.974)
Naïve Bayes	0.842	0.842	0.842	0.842	0.950 (0.939, 0.961)
Logistic regression	0.684	0.684	0.684	0.684	0.910 (0.898, 0.920)

**TABLE 6:** The performance evaluation results of the six models in the “other days” classification.

Models	Accuracy	Precision	Recall rate	F‐measure	AUC (95% CI)
Decision tree	0.944	0.944	0.944	0.944	0.950 (0.948, 0.960)
Random forest	0.943	0.943	0.943	0.943	0.940 (0.934, 0.949)
Naïve Bayes	0.942	0.942	0.942	0.942	0.940 (0.931, 0.944)
Logistic regression	0.944	0.944	0.944	0.944	0.930 (0.926, 0.941)
KNN	0.944	0.944	0.944	0.944	0.920 (0.910, 0.928)
SVM	0.943	0.943	0.943	0.943	0.900 (0.891, 0.912)

### 3.4. Output of the DT Model for Patient Classification

**FIGURE 5:**
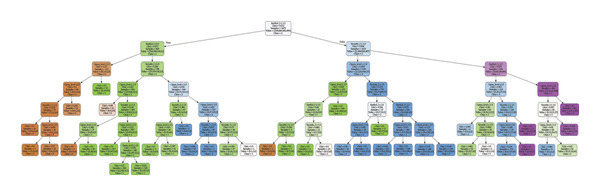
Visualization of the decision tree model for patient classification in “the day of surgery and first postoperative day” group.

**FIGURE 6:**
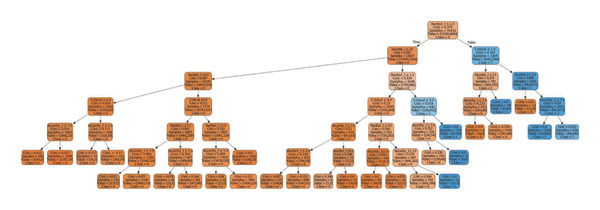
Visualization of the decision tree model for patient classification in the “other days” group.

The DT model was ultimately selected for the development of patient classification. The classification output processes of patients on “the day of surgery and the first postoperative day” and “other days” are shown in Figures 5 and 6, respectively.

### 3.5. Prospective Temporal Validation

**FIGURE 7:**
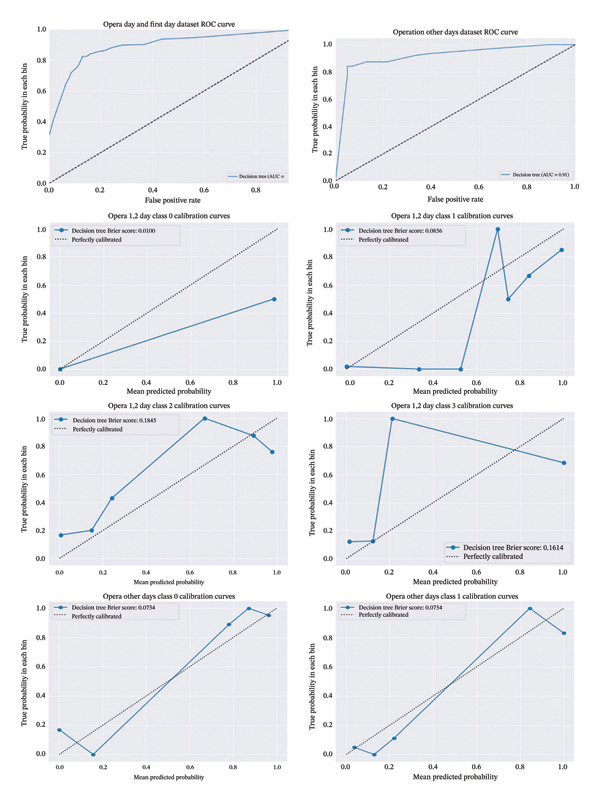
ROC and calibration curves for external validation of the decision tree model.

The DT model demonstrated consistent classification performance. For patients in the “day of surgery and first postoperative day” group, all performance metrics ranged from 0.753 to 0.892, while for the “other days” group, values ranged from 0.915 to 0.924. The Brier scores varied between 0.0100 and 0.1845, confirming acceptable calibration (Table 7, Figure 7).

**TABLE 7:** Performance indicators for external validation of the decision tree model.

Classification	Accuracy	Precision	Recall rate	F‐measure	AUC (95% CI)
“The day of surgery and first postoperative day”	0.753	0.753	0.753	0.753	0.892 (0.875, 0.951)
“Other days”	0.924	0.924	0.924	0.924	0.915 (0.865, 0.955)

### 3.6. Assessment of Model Clinical Accuracy

A total of 357 patients were included in the prospective temporal validation cohort. Category 1 contained the largest proportion of patients (201 cases, 56.30%). The overall prediction accuracy for NHPPD was 89.08%. The accuracy was highest in Category 1 (96.51%) and lowest in Category 4 (73.68%) (Table 8).

**TABLE 8:** The patient classification model’s prediction accuracy rate of the NHPPD (*n* = 357).

Category	Total number	The result is within the prediction range (*n*)	The result is outside the prediction range (*n*)	Prediction accuracy (%)
1	201	194	7	96.51
2	63	52	11	81.25
Surgery‐1	41	31	10	75.61
Surgery‐2	33	27	6	82.35
Surgery‐3	19	14	5	73.68
Total	357	318	39	89.08

### 3.7. Final PCS

The NHPPD estimates for each patient category were adjusted according to the observed nursing workload in routine clinical practice and the external validation results. The finalized PCS and corresponding nursing time requirements are presented in Tables 9 and 10.

**TABLE 9:** Details of “the day of surgery and first postoperative day” patient classification (revised).

Category	Surgery level	Self‐care ability	Severity of illness	NHPPD (min)
Surgery ‐1	1	Mild dependence	Severe	99.82
	2	Mild dependence	Moderate/severe	
		Moderate dependence	Mild/moderate/severe/critical	
		Severe dependence	Moderate/severe	
	3	Mild dependence	Mild/moderate	
	4	No dependence	Moderate	
		Mild dependence	Mild/moderate/severe/critical	
		Severe dependence	Mild	

Surgery‐2	3	Mild dependence	Severe	169.36
		Moderate dependence	Mild/moderate/severe	
		Severe dependence	Moderate	
	4	Moderate dependence	Mild/moderate/severe	
		Severe dependence	Moderate	

Surgery‐3	3	Moderate dependence	Critical	248.88
		Severe dependence	Severe/critical	
	4	Moderate dependence	Severe	
		Severe dependence	Severe/critical	

*Note:* Surgery level: 1 = primary surgery; 2 = secondary surgery; 3 = tertiary surgery; 4 = quaternary surgery; self‐care ability: no dependence = 100 scores; mild dependence = 61–99 scores; moderate dependence = 41–60 scores; severe dependence: ≤ 40 scores. Severity of illness: mild = 0∼1 score; moderate: 2∼6 scores; severe: 7∼11 scores; critical: ≥ 12 scores.

**TABLE 10:** Details of the “other days” patient classification (revised).

Category	Whether critically ill	Self‐care ability	Severity of illness	NHPPD (min)
1	No	No dependence	Mild/moderate/severe/critical	57.40
		Mild dependence	Mild/moderate/severe/critical	
		Moderate dependence	Mild/moderate/severe	
		Severe dependence	Mild/moderate	
	Yes	No dependence	Mild/moderate/severe/critical	
		Mild dependence	Mild/moderate/severe	
		Moderate dependence	Mild/moderate	
		Severe dependence	Mild	

2	No	Moderate dependence	Critical	205.30
		Severe dependence	Severe/critical	
	Yes	Mild dependence	Critical	
		Moderate dependence	Severe/critical	
		Severe dependence	Moderate/severe/critical	

*Note:* Self‐care ability: no dependence = 100 score; mild dependence = 61‒99 score; moderate dependence = 41‒60 scores; severe dependence: ≤ 40 score; severity of illness: mild = 0‐1 score; moderate: 2‒6 scores; severe: 7‒11 scores; critical: ≥ 12 scores.

## 4. Discussion

This study developed and validated a gastroenterology‐specific PCS using a DT‐based machine learning approach to improve the objectivity and accuracy of nursing workload estimation. Recent literature highlights that the integration of digital technology into nursing practice is fundamentally reshaping care models [20]. Machine learning–based PCS can therefore be used not only as workload stratification tools but also as a core component of intelligent, responsive, and personalized nurse staffing pathways. By linking algorithmic outputs to clinical decisions, the present PCS supports the shift from reactive, episode‐based care toward proactive, continuous, and data‐driven nursing practice. The final PCS consisted of two major patient groups and five subcategories, each defined by specific clinical indicators and associated 24‐hour nursing time requirements.

A key finding was the pronounced difference in nursing workload between the “day of surgery and first postoperative day” and all subsequent hospital day groups. Patients required an average of 118.16 min of direct nursing care during the perioperative period, compared with only 60.89 min on other days (*Z* = −38.799, *p* < 0.001). This pattern aligns with the findings of prior studies demonstrating that nursing demand peaks within the first 24 h after surgery [21]. For example, research in surgical intensive care units reported that 82.4% of total nursing workload occurred during the first postoperative day [22]. Consistent with the approaches of Shu‐Qi [23] and Xin‐Rui [24], we therefore constructed separate PCS models for these two time periods to improve the precision of classification. This method of stratifying and classifying patients also aligns with earlier findings and highlights the complexity of clinical monitoring and the necessity of using integrated approaches [25].

The PCS was further refined by the application of integer‐based workload thresholds to classify patient acuity. For patients on the day of surgery and first postoperative day, NHPPD was divided into four strata (≤ 1, 1‐2, 2‐3, and > 3 h). For patients in the other day group, over 80% had NHPPD ≤ 2 h, leading to the use of only two categories (≤ 2 h and > 2 h). This approach is consistent with previous studies on PCS development, both national and international [26–31], although the specific cutoffs vary across settings. After hyperparameterization, all six machine learning models achieved strong performance, with accuracies above 0.80 and AUC values ranging from 0.910 to 0.980 for perioperative patients and accuracies of 0.942–0.944 and AUC values of 0.900–0.950 for patients on other days. External validation further showed an overall NHPPD prediction accuracy of 89.08%, confirming that the time‐based classification scheme reflected the real clinical workload. These results demonstrate that the PCS had a solid theoretical grounding, aligned with empirical data, and offered reliable support for nursing staff allocation in gastroenterology wards.

The analysis demonstrated that disease severity, self‐care capacity, surgical grade, and critical illness status were all independent determinants of the time spent on 24‐hour direct nursing (all *p* < 0.001) and were strongly and positively associated with nursing workload. These findings are consistent with prior research showing that greater clinical severity [32–34], poorer self‐care ability [35, 36], and higher surgical complexity [37–39] substantially increased nursing time requirements. In this study, disease severity and functional status were assessed using validated objective instruments, including the Disease Severity Evaluation Scale [17] and the Barthel Index [40], thereby strengthening the scientific rigor of the classification. In addition, the inclusion of critical illness status [41], a reflection of the need for intensive monitoring and specialized nursing, and surgical grade [42, 43] further enhanced the clinical relevance of the system. Collectively, the incorporation of the indicators of medical condition (disease severity and surgical grade), functional status (self‐care ability), and care intensity (critical illness) enabled a more comprehensive and accurate estimation of nursing workload and provided a robust evidence base for patient classification. Based on these principles, the final PCS included two major patient groups and five subcategories. Category 1 included patients on the day of surgery and the first postoperative day. These patients are further divided into three subgroups according to surgical level, self‐care ability, and disease severity (Surgery 1, Surgery 2, and Surgery 3), with corresponding 24‐hour nursing time requirements of 1.66, 2.82, and 4.15 h, respectively. Category 2 comprised patients hospitalized on all other days and was divided into two subgroups based on critical illness status, self‐care ability, and disease severity (Category 1 and Category 2), requiring 0.96 and 3.42 nursing hours over 24 h, respectively. Both internal and prospective temporal validation confirmed that the model had good fit and satisfactory predictive performance.

From an organizational perspective, the integration of systematic assessment tools has been shown to play a critical role in enhancing the quality of nursing care [44]. By providing a standardized, data‐driven approach to patient classification, this proposed machine learning–based system can support nursing management in providing a more accurate prediction of workload, allocation of staffing resources, and identification of patients with complex care needs. Such systematic assessments enable organizations to move beyond subjective or experience‐based judgments, thereby reducing variability in care delivery and improving both efficiency and patient safety. Furthermore, embedding this system into routine clinical workflows could facilitate continuous monitoring of nursing care needs and support evidence‐based decision‐making at the institutional level. Future research should therefore examine the implementation of this system in real‐world organizational settings and assess its impact on measurable nursing quality outcomes, such as patient adverse events, nurse staffing adequacy, and care timeliness.

The application of machine learning to PCS development in this study introduced a novel, objective, and data‐driven approach to the prediction of nursing workload. Compared with traditional linear or experience‐based classification methods, this machine learning approach offers superior precision and clinical relevance. Nevertheless, the study has several limitations. Firstly, as the model was derived from and validated exclusively on data from a single institutional cohort, its external generalizability may be limited, and this preliminary performance does not fully address potential overfitting or guarantee generalizability. Secondly, the retrospective data collection in the first study component may have missed undocumented but delivered nursing care, potentially biasing workload estimates. Finally, we acknowledge that psychosocial aspects, although not addressed in the current model, are highly relevant to clinical practice. As psychosocial factors have been shown to be closely related to nursing care needs and workloads [45], the incorporation of these dimensions represents an important direction for future research. Future multicenter prospective studies with larger and more contemporaneous samples are warranted to further validate and refine the system, thereby enhancing its robustness and broader clinical utility.

## 5. Implications for Nursing Management

This study provides a practical and innovative staffing tool for gastroenterology wards. When integrated with intelligent nursing information systems and electronic medical records, the PCS can automatically capture patient characteristics and estimate the required nursing time, thereby enabling nurse managers to calculate staffing needs more accurately. Moreover, the PCS can be combined with nursing management frameworks to dynamically match nurse competencies with patient acuity, facilitating precise nurse–patient assignment, enhancing workforce efficiency, and ultimately supporting the delivery of high‐quality care. However, as it is possible that NHPPD models may underestimate the time nurses spend in the provision of patient care, nursing managers should interpret their outputs with caution and make unit‐specific adjustments based on the actual clinical and operational circumstances. Finally, this PCS could also be combined with remote medical services and remote monitoring tools, enabling dynamic workload prediction and guiding dynamic scheduling of nursing staff, thereby enhancing the efficiency of the utilization of nursing human resources.

## 6. Conclusions

The machine learning–based PCS developed for the gastroenterology ward enabled rapid classification of patients using objective clinical indicators and may facilitate reasonably accurate prediction of 24‐hour nursing time requirements. This tool represents a potentially scientifically grounded and operationally feasible approach for quantifying nursing workload and guiding staffing decisions. In addition, the findings may contribute to the theoretical and methodological frameworks for PCS development and offer preliminary evidence that could inform workforce planning and policy formulation aimed at improving efficiency and quality in public hospitals. Nevertheless, further validation in different settings and larger patient populations is needed before broader generalizability can be assumed.

## Funding

This study was supported by the National Natural Science Foundation of China (grant no. 72174135), West China Nursing Discipline Development Special Found Project, Sichuan University (grant no. HXHL20049), and Sichuan Province Science and Technology Support Program (grant no. 2024JDKP0097).

## Ethics Statement

This study was approved by the West China Hospital of Sichuan University (approval number: 2021‐444).

## Conflicts of Interest

The authors declare no conflicts of interest.

## Data Availability

The datasets consulted during this study are available from the corresponding authors on reasonable request.
